# Crystal structure of diethyl 3,3′-[(2,4-di­chloro­phen­yl)methyl­idene]bis­(1*H*-indole-2-carboxyl­ate)

**DOI:** 10.1107/S2056989017015730

**Published:** 2017-11-03

**Authors:** Yu-Long Li, Hong-Shun Sun, Hong Jiang, Yu-Liang Chen, Yang-Feng Chen

**Affiliations:** a Targeted MRI Contrast Agents Laboratory of Jiangsu Province, Nanjing Polytechnic Institute, Geguan Road No. 265 Nanjing, Nanjing 210048, People’s Republic of China

**Keywords:** crystal structure, bis­indole, MRI, contrast agent

## Abstract

In the title compound, C_29_H_24_Cl_2_N_2_O_4_, the mean planes of the two indole ring systems (r.m.s. deviations = 0.1249 and 0.0075 Å) are approximately perpendic­ular to one another, with a dihedral angle of 80.9 (5)° between them. The benzene ring is inclined to the mean planes of the two indole ring systems by 76.1 (3) and 78.3 (4)°. Weak intra­molecular C—H⋯π inter­actions affect the mol­ecular conformation. In the crystal, pairs of N—H⋯O hydrogen bonds link the mol­ecules into inversion dimers which are further linked into supra­molecular chains by N—H⋯O hydrogen bonds and short Cl—Cl contacts.

## Abstract 2

In the title compound, the two indole ring systems are approximately perpendicular to one another, making a dihedral angle of 80.9 (5)°. In the crystal, pairs of N—H⋯O hydrogen bonds link the mol­ecules into inversion dimers and these are further linked by N—H⋯O and hydrogen bonds and short Cl—Cl contacts into supra­molecular chains.

## Chemical context   

Bis(indol­yl)methane derivatives are abundantly present in various terrestrial and marine natural resources (Porter *et al.*,1977 ▸; Sundberg, 1996 ▸). They are important anti­biotics in the field of pharmaceuticals with diverse activities, displaying anti­cancer, anti­leishmanial and anti­hyperlipidemic properties (Chang *et al.*, 1999 ▸; Ge *et al.*, 1999 ▸). Furthermore, bis­(indolyl)methane derivatives can also be used as precursors for MRI necrosis avid contrast agents (Ni, 2008 ▸). In recent years, we have reported the synthesis and crystal structures of some similar bis­(indoly)methane compounds (Sun *et al.*, 2012 ▸, 2015 ▸; Li *et al.*, 2014 ▸; Lu *et al.*, 2014 ▸). We report here the mol­ecular and crystal structure of the title bis­(indoly)methane derivative.
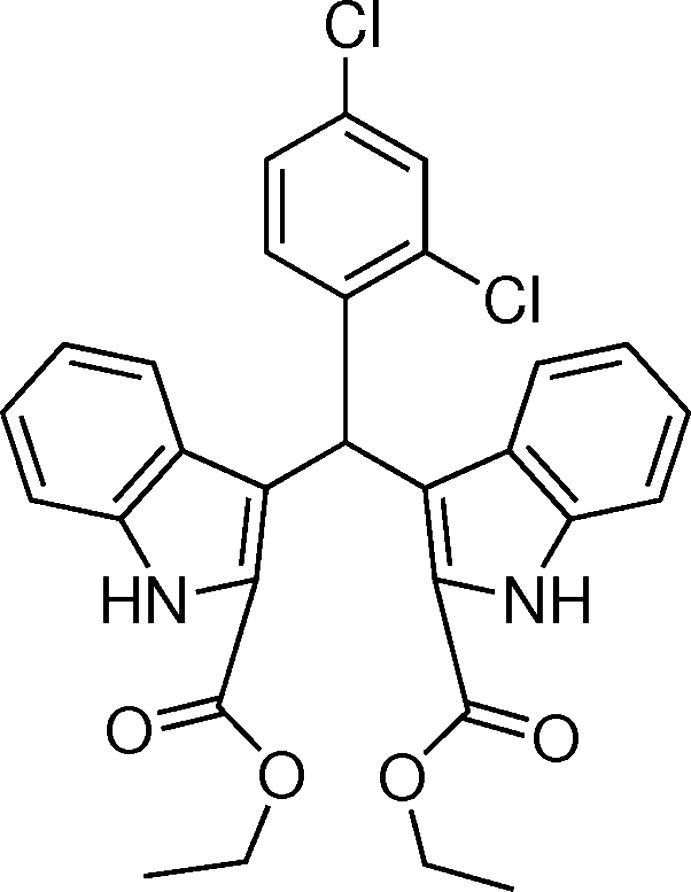


## Structural commentary   

**Figure 1:**
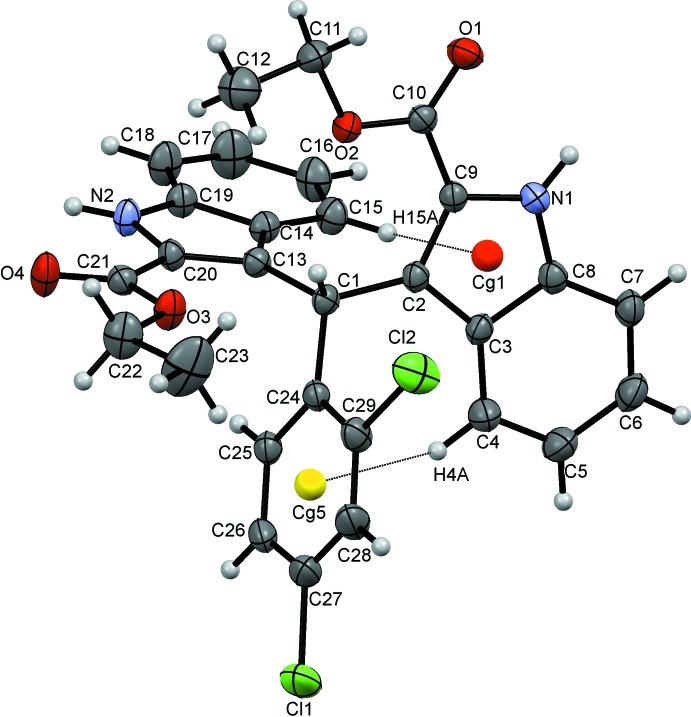
The mol­ecular structure of the title mol­ecule showing the atom-labelling scheme. Displacement ellipsoids are drawn at the 30% probability level. Intra­molecular C—H⋯π(ring) contacts (Table 1 ▸) are shown as dotted black lines with ring centroids displayed as coloured spheres.

The mol­ecular structure of the title compound is shown in Fig. 1 ▸. The overall conformation of the mol­ecule is affected by intra­molecular C4—H4*A*⋯*Cg*5 and C15—H15*A*⋯*Cg*1 inter­actions (*Cg*1 and *Cg*5 are the centroids of the N1,C8,C3,C2,C9 and C24–C29 rings, respectively), Fig. 1 ▸, Table 1 ▸. The two indole ring systems are nearly perpendicular to one another, subtending a dihedral angle of 80.9 (5)° while the C24–C29 benzene ring is inclined to the N1/C2–C9 and N2/C13–C20 indole ring systems by dihedral angles of 76.1 (3) and 78.3 (4)°, respectively. The carboxyl groups lie close to the planes of the indole ring systems to which they are bound, with dihedral angles between the carboxyl groups and the mean planes of the N1/C2–C9 and N2/C13–C20 indole ring systems of 8.3 (5) and 5.6 (3)°, respectively.

## Supra­molecular features   

**Figure 2:**
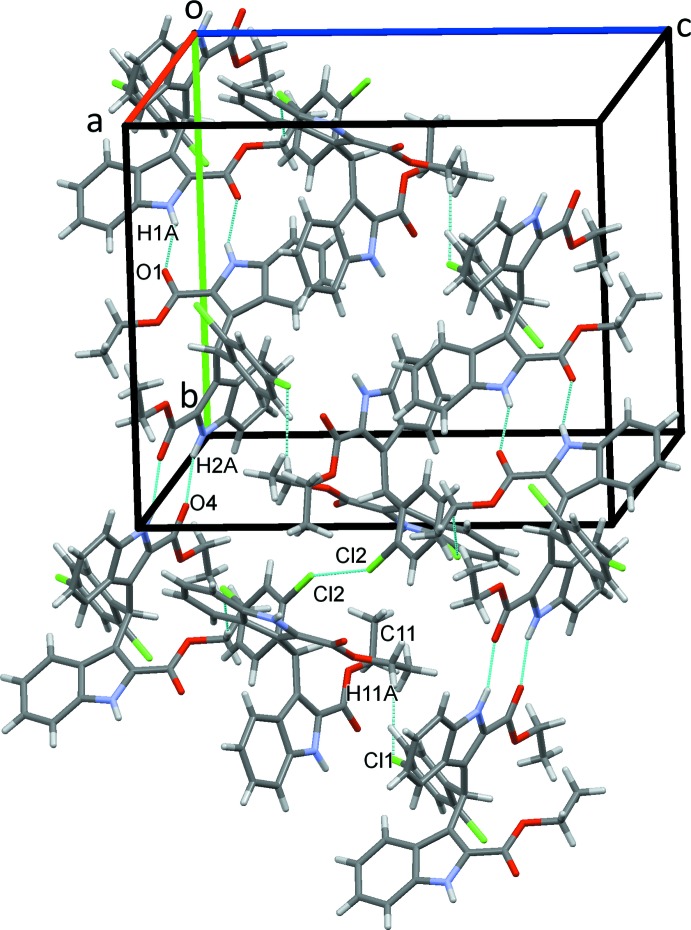
A packing diagram of the title compound. Hydrogen bonds (Table 1 ▸) and Cl⋯Cl contacts are shown as dashed lines.

In the crystal, pairs of N1—H1*A*⋯O1 and N2—H2*A*⋯O4 hydrogen bonds, Table 1 ▸, link the mol­ecules into inversion dimers that form supramolecular chains along the *b*-axis direction. C11—H11*A*⋯Cl1 and short Cl2⋯Cl2 contacts [Cl2⋯Cl2(1 − *x*, 1 − *y*, −*z*) = 3.467 (2) Å] bridge these chains and form sheets of mol­ecules parallel to (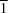
12), Fig. 2 ▸.

## Database survey   

Several similar structures have been reported previously, *i.e*. diethyl 3,3′-(phenyl­methyl­ene)bis­(1*H*-indole-2-carboxyl­ate) (Sun *et al.*, 2012 ▸), dimethyl 3,3′-[(4-fluoro­phen­yl)methyl­ene]bis­(1*H*-indole-2-carboxyl­ate) (Sun *et al.*, 2015 ▸) dimethyl 3,3′-[(4-chloro­phen­yl) methyl­ene]bis­(1*H*-indole-2-carboxyl­ate) (Li *et al.*, 2014 ▸) and dimethyl 3,3′-[(3-fluoro­phen­yl)methyl­ene]bis­(1*H*-indole-2-carboxyl­ate) (Lu *et al.*, 2014 ▸). In these structures, the two indole ring systems are also nearly perpendicular to one another, making dihedral angles of 82.0 (5), 84.0 (5), 79.5 (4) and 87.8 (5)°, respectively.

## Synthesis and crystallization   

Ethyl indole-2-carboxyl­ate (1.88 g, 10 mmol) was dissolved in 20 ml ethanol; commercially available 2,4-di­chloro­benzalde­hyde (0.88 g, 5 mmol) was added and the mixture was heated to reflux temperature. Concentrated HCl (0.5 ml) was added and the reaction was left for 1 h. After cooling, the white product was filtered off and washed thoroughly with ethanol. The reaction was monitored with TLC (AcOEt:hexane = 1:3). Colourless block-like crystals of the title compound suitable for X-ray analysis were obtained in 92% yield by slow evaporation of an ethanol solution.

## Refinement   

Crystal data, data collection and structure refinement details are summarized in Table 2 ▸. H atoms were positioned geometrically with N—H = 0.86 Å and C—H = 0.93–0.98 Å, and constrained to ride on their parent atoms with *U*_iso_(H) = *xU*_eq_(C,N), where *x* = 1.5 for methyl H atoms and 1.2 for all others.

## Supplementary Material

Crystal structure: contains datablock(s) I. DOI: 10.1107/S2056989017015730/sj5539sup1.cif

Structure factors: contains datablock(s) I. DOI: 10.1107/S2056989017015730/sj5539Isup2.hkl

Supporting information file. DOI: 10.1107/S2056989017015730/sj5539Isup3.cml

CCDC reference: 1582719

Additional supporting information:  crystallographic information; 3D view; checkCIF report

## Figures and Tables

**Table 1:** Hydrogen-bond geometry (Å, °) *Cg*1 and *Cg*5 are the centroids of the N1,C8,C3,C2,C9 and C24–C29 rings respectively.

*D*—H⋯*A*	*D*—H	H⋯*A*	*D*⋯*A*	*D*—H⋯*A*
N1—H1*A*⋯O1^i^	0.86	2.07	2.864 (4)	152
N2—H2*A*⋯O4^ii^	0.86	2.04	2.871 (4)	161
C11—H11*A*⋯Cl1^iii^	0.97	2.81	3.731 (5)	158
C4—H4*A*⋯*Cg*5	0.93	2.77	3.516 (4)	137
C15—H15*A*⋯*Cg*1	0.93	2.72	3.476 (5)	139

Symmetry codes: (i) 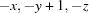; (ii) 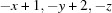; (iii) 
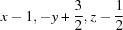
.

**Table 2:** Experimental details

Crystal data	
Chemical formula	C_29_H_24_Cl_2_N_2_O_4_
*M*_r_	535.40
Crystal system, space group	Monoclinic, *P*2_1_/*c*
Temperature (K)	293
*a*, *b*, *c* (Å)	9.776 (2), 15.939 (3), 17.581 (4)
β (°)	101.94 (3)
*V* (Å^3^)	2680.2 (9)
*Z*	4
Radiation type	Mo *K*α
μ (mm^−1^)	0.28
Crystal size (mm)	0.30 × 0.20 × 0.10

Data collection	
Diffractometer	Enraf–Nonius CAD-4
Absorption correction	ψ scan (North *et al.*, 1968 ▸)
*T*_min_, *T*_max_	0.921, 0.973
No. of measured, independent and observed [*I* > 2σ(*I*)] reflections	5221, 4917, 2864
*R*_int_	0.034
(sin θ/λ)_max_ (Å^−1^)	0.603

Refinement	
*R*[*F* ^2^ > 2σ(*F* ^2^)], *wR*(*F* ^2^), *S*	0.067, 0.192, 1.00
No. of reflections	4917
No. of parameters	328
H-atom treatment	H-atom parameters constrained
Δρ_max_, Δρ_min_ (e Å^−3^)	0.69, −1.14

Computer programs: *CAD-4 EXPRESS* (Enraf–Nonius, 1994 ▸), *XCAD4* (Harms & Wocadlo, 1995 ▸), *SHELXTL* (Sheldrick, 2008 ▸) and *Mercury* (Macrae *et al.*, 2008 ▸).

## supplementary crystallographic information

### Crystal data

**Table:**

C_29_H_24_Cl_2_N_2_O_4_	*F*(000) = 1112
*M**_r_* = 535.40	*D*_x_ = 1.327 Mg m^−^^3^
Monoclinic, *P*2_1_/*c*	Mo *K*α radiation, λ = 0.71073 Å
Hall symbol: -P 2ybc	Cell parameters from 25 reflections
*a* = 9.776 (2) Å	θ = 9–13°
*b* = 15.939 (3) Å	µ = 0.28 mm^−^^1^
*c* = 17.581 (4) Å	*T* = 293 K
β = 101.94 (3)°	Block, colorless
*V* = 2680.2 (9) Å^3^	0.30 × 0.20 × 0.10 mm
*Z* = 4	

### Data collection

**Table:**

Enraf–Nonius CAD-4 diffractometer	2864 reflections with *I* > 2σ(*I*)
Radiation source: fine-focus sealed tube	*R*_int_ = 0.034
Graphite monochromator	θ_max_ = 25.4°, θ_min_ = 1.7°
ω/2θ scans	*h* = 0→11
Absorption correction: ψ scan (North *et al.*, 1968)	*k* = 0→19
*T*_min_ = 0.921, *T*_max_ = 0.973	*l* = −21→21
5221 measured reflections	3 standard reflections every 200 reflections
4917 independent reflections	intensity decay: 1%

### Refinement

**Table:**

Refinement on *F*^2^	Primary atom site location: structure-invariant direct methods
Least-squares matrix: full	Secondary atom site location: difference Fourier map
*R*[*F*^2^ > 2σ(*F*^2^)] = 0.067	Hydrogen site location: inferred from neighbouring sites
*wR*(*F*^2^) = 0.192	H-atom parameters constrained
*S* = 1.00	*w* = 1/[σ^2^(*F*_o_^2^) + (0.1*P*)^2^ + 0.670*P*] where *P* = (*F*_o_^2^ + 2*F*_c_^2^)/3
4917 reflections	(Δ/σ)_max_ < 0.001
328 parameters	Δρ_max_ = 0.69 e Å^−^^3^
0 restraints	Δρ_min_ = −1.13 e Å^−^^3^

### Special details

**Table:**

Geometry. All esds (except the esd in the dihedral angle between two l.s. planes) are estimated using the full covariance matrix. The cell esds are taken into account individually in the estimation of esds in distances, angles and torsion angles; correlations between esds in cell parameters are only used when they are defined by crystal symmetry. An approximate (isotropic) treatment of cell esds is used for estimating esds involving l.s. planes.
Refinement. Refinement of F^2^ against ALL reflections. The weighted R-factor wR and goodness of fit S are based on F^2^, conventional R-factors R are based on F, with F set to zero for negative F^2^. The threshold expression of F^2^ > 2sigma(F^2^) is used only for calculating R-factors(gt) etc. and is not relevant to the choice of reflections for refinement. R-factors based on F^2^ are statistically about twice as large as those based on F, and R- factors based on ALL data will be even larger.

### Fractional atomic coordinates and isotropic or equivalent isotropic displacement parameters (Å^2^)

**Table:**

	*x*	*y*	*z*	*U*_iso_*/*U*_eq_	
Cl1	0.99326 (13)	0.65116 (9)	0.32372 (10)	0.0997 (6)	
O1	0.0795 (3)	0.57429 (17)	−0.06706 (15)	0.0537 (7)	
N1	0.1566 (3)	0.53608 (18)	0.09007 (17)	0.0440 (8)	
H1A	0.0821	0.5101	0.0675	0.053*	
C1	0.4458 (4)	0.6877 (2)	0.0923 (2)	0.0371 (8)	
H1B	0.4656	0.6719	0.0418	0.044*	
Cl2	0.61986 (13)	0.53457 (7)	0.08076 (8)	0.0813 (5)	
O2	0.2675 (3)	0.65654 (16)	−0.05724 (14)	0.0478 (7)	
N2	0.3672 (3)	0.91230 (18)	0.04635 (18)	0.0458 (8)	
H2A	0.3841	0.9599	0.0272	0.055*	
C2	0.3421 (4)	0.6237 (2)	0.1094 (2)	0.0373 (8)	
O3	0.6370 (3)	0.77571 (16)	0.01095 (16)	0.0514 (7)	
C3	0.3369 (4)	0.5821 (2)	0.1812 (2)	0.0400 (8)	
O4	0.6115 (3)	0.91368 (16)	−0.00828 (18)	0.0600 (8)	
C4	0.4188 (4)	0.5829 (3)	0.2572 (2)	0.0554 (11)	
H4A	0.4952	0.6187	0.2702	0.066*	
C5	0.3842 (5)	0.5301 (3)	0.3120 (3)	0.0628 (12)	
H5A	0.4388	0.5304	0.3620	0.075*	
C6	0.2703 (5)	0.4761 (3)	0.2950 (3)	0.0598 (12)	
H6A	0.2510	0.4410	0.3337	0.072*	
C7	0.1858 (4)	0.4738 (2)	0.2225 (2)	0.0526 (10)	
H7A	0.1089	0.4382	0.2111	0.063*	
C8	0.2203 (4)	0.5274 (2)	0.1659 (2)	0.0424 (9)	
C9	0.2289 (3)	0.5925 (2)	0.0549 (2)	0.0379 (8)	
C10	0.1837 (4)	0.6072 (2)	−0.0283 (2)	0.0378 (8)	
C11	0.2406 (5)	0.6680 (3)	−0.1409 (2)	0.0611 (12)	
H11A	0.1587	0.7029	−0.1577	0.073*	
H11B	0.2245	0.6142	−0.1670	0.073*	
C12	0.3646 (5)	0.7087 (3)	−0.1591 (3)	0.080	
H12A	0.3509	0.7169	−0.2143	0.120*	
H12B	0.4449	0.6737	−0.1418	0.120*	
H12C	0.3789	0.7619	−0.1333	0.120*	
C13	0.3873 (3)	0.7766 (2)	0.0822 (2)	0.0367 (8)	
C14	0.2657 (4)	0.8105 (2)	0.1040 (2)	0.0393 (8)	
C15	0.1617 (4)	0.7797 (3)	0.1419 (2)	0.0499 (10)	
H15A	0.1632	0.7241	0.1582	0.060*	
C16	0.0581 (5)	0.8334 (3)	0.1541 (3)	0.0624 (12)	
H16A	−0.0111	0.8133	0.1786	0.075*	
C17	0.0542 (5)	0.9170 (3)	0.1308 (3)	0.0755 (14)	
H17A	−0.0165	0.9517	0.1409	0.091*	
C18	0.1515 (4)	0.9490 (3)	0.0938 (3)	0.0610 (12)	
H18A	0.1474	1.0047	0.0776	0.073*	
C19	0.2575 (4)	0.8959 (2)	0.0809 (2)	0.0430 (9)	
C20	0.4467 (4)	0.8411 (2)	0.0468 (2)	0.0378 (8)	
C21	0.5712 (4)	0.8480 (2)	0.0141 (2)	0.0410 (9)	
C22	0.7623 (5)	0.7796 (3)	−0.0215 (3)	0.0673 (13)	
H22A	0.8306	0.8168	0.0093	0.081*	
H22B	0.7397	0.8006	−0.0744	0.081*	
C23	0.8196 (6)	0.6929 (4)	−0.0202 (4)	0.113 (2)	
H23A	0.9022	0.6932	−0.0417	0.170*	
H23B	0.7509	0.6566	−0.0505	0.170*	
H23C	0.8427	0.6730	0.0325	0.170*	
C24	0.5846 (4)	0.6817 (2)	0.1501 (2)	0.0391 (8)	
C25	0.6324 (4)	0.7431 (2)	0.2053 (2)	0.0437 (9)	
H25A	0.5790	0.7912	0.2068	0.052*	
C26	0.7582 (4)	0.7340 (3)	0.2582 (2)	0.0530 (11)	
H26A	0.7885	0.7758	0.2948	0.064*	
C27	0.8370 (4)	0.6642 (3)	0.2566 (3)	0.0614 (12)	
C28	0.7963 (4)	0.6021 (3)	0.2019 (3)	0.0634 (12)	
H28A	0.8513	0.5547	0.2006	0.076*	
C29	0.6715 (4)	0.6124 (2)	0.1493 (2)	0.0505 (10)	

### Atomic displacement parameters (Å^2^)

**Table:**

	*U*^11^	*U*^22^	*U*^33^	*U*^12^	*U*^13^	*U*^23^
Cl1	0.0617 (8)	0.0762 (9)	0.1361 (13)	−0.0114 (7)	−0.0373 (8)	0.0215 (8)
O1	0.0412 (15)	0.0659 (18)	0.0512 (16)	−0.0065 (14)	0.0034 (13)	0.0008 (14)
N1	0.0394 (17)	0.0439 (18)	0.0467 (18)	−0.0081 (14)	0.0045 (14)	0.0025 (14)
C1	0.0398 (19)	0.0283 (17)	0.043 (2)	0.0036 (15)	0.0076 (16)	−0.0009 (15)
Cl2	0.0746 (8)	0.0529 (7)	0.1067 (10)	0.0220 (6)	−0.0039 (7)	−0.0310 (7)
O2	0.0522 (15)	0.0472 (15)	0.0421 (15)	−0.0103 (13)	0.0050 (12)	0.0081 (12)
N2	0.0482 (18)	0.0284 (15)	0.064 (2)	0.0026 (14)	0.0178 (16)	0.0064 (14)
C2	0.0386 (19)	0.0289 (18)	0.044 (2)	−0.0002 (15)	0.0090 (17)	0.0012 (15)
O3	0.0497 (16)	0.0397 (14)	0.0711 (18)	0.0091 (12)	0.0271 (14)	0.0049 (13)
C3	0.044 (2)	0.0314 (18)	0.044 (2)	0.0024 (16)	0.0090 (17)	0.0068 (16)
O4	0.0536 (17)	0.0404 (15)	0.094 (2)	0.0019 (13)	0.0339 (16)	0.0158 (15)
C4	0.058 (3)	0.056 (2)	0.050 (2)	−0.012 (2)	0.005 (2)	0.005 (2)
C5	0.061 (3)	0.070 (3)	0.054 (3)	−0.003 (2)	0.005 (2)	0.018 (2)
C6	0.063 (3)	0.057 (3)	0.062 (3)	0.001 (2)	0.019 (2)	0.023 (2)
C7	0.050 (2)	0.047 (2)	0.063 (3)	−0.0070 (19)	0.017 (2)	0.011 (2)
C8	0.039 (2)	0.036 (2)	0.053 (2)	0.0003 (16)	0.0113 (17)	0.0015 (17)
C9	0.0347 (18)	0.0327 (18)	0.047 (2)	−0.0003 (15)	0.0107 (16)	0.0019 (16)
C10	0.0344 (19)	0.0329 (18)	0.045 (2)	0.0045 (16)	0.0061 (17)	−0.0003 (16)
C11	0.058 (3)	0.068 (3)	0.052 (3)	−0.007 (2)	−0.002 (2)	0.016 (2)
C12	0.080	0.080	0.080	0.000	0.017	0.000
C13	0.0364 (19)	0.0316 (18)	0.042 (2)	0.0024 (15)	0.0072 (16)	−0.0020 (15)
C14	0.040 (2)	0.0365 (19)	0.043 (2)	0.0038 (16)	0.0105 (16)	−0.0017 (16)
C15	0.046 (2)	0.050 (2)	0.058 (3)	0.0015 (19)	0.021 (2)	0.0061 (19)
C16	0.059 (3)	0.059 (3)	0.078 (3)	0.000 (2)	0.035 (2)	0.002 (2)
C17	0.064 (3)	0.058 (3)	0.114 (4)	0.021 (2)	0.042 (3)	−0.001 (3)
C18	0.056 (3)	0.041 (2)	0.093 (3)	0.012 (2)	0.030 (2)	0.006 (2)
C19	0.040 (2)	0.038 (2)	0.054 (2)	0.0029 (17)	0.0158 (18)	0.0010 (17)
C20	0.0388 (19)	0.0288 (18)	0.046 (2)	−0.0016 (15)	0.0097 (16)	−0.0008 (15)
C21	0.039 (2)	0.039 (2)	0.045 (2)	−0.0015 (17)	0.0076 (17)	0.0001 (17)
C22	0.063 (3)	0.061 (3)	0.089 (3)	0.012 (2)	0.041 (3)	0.002 (2)
C23	0.102 (4)	0.099 (5)	0.158 (6)	0.055 (4)	0.071 (4)	0.029 (4)
C24	0.038 (2)	0.0334 (18)	0.046 (2)	−0.0048 (16)	0.0095 (16)	0.0011 (16)
C25	0.045 (2)	0.0338 (19)	0.052 (2)	−0.0043 (17)	0.0096 (18)	0.0023 (17)
C26	0.060 (3)	0.043 (2)	0.052 (2)	−0.018 (2)	0.004 (2)	0.0031 (18)
C27	0.045 (2)	0.052 (3)	0.080 (3)	−0.008 (2)	−0.006 (2)	0.016 (2)
C28	0.042 (2)	0.046 (2)	0.097 (4)	0.005 (2)	0.001 (2)	0.008 (2)
C29	0.046 (2)	0.034 (2)	0.069 (3)	0.0024 (18)	0.006 (2)	−0.0040 (19)

### Geometric parameters (Å, º)

**Table:**

Cl1—C27	1.738 (4)	C11—H11B	0.9700
O1—C10	1.221 (4)	C12—H12A	0.9600
N1—C8	1.356 (5)	C12—H12B	0.9600
N1—C9	1.368 (4)	C12—H12C	0.9600
N1—H1A	0.8600	C13—C20	1.389 (5)
C1—C2	1.511 (5)	C13—C14	1.429 (5)
C1—C24	1.521 (5)	C14—C15	1.414 (5)
C1—C13	1.525 (4)	C14—C19	1.418 (5)
C1—H1B	0.9800	C15—C16	1.377 (5)
Cl2—C29	1.730 (4)	C15—H15A	0.9300
O2—C10	1.312 (4)	C16—C17	1.392 (6)
O2—C11	1.450 (5)	C16—H16A	0.9300
N2—C19	1.363 (4)	C17—C18	1.358 (6)
N2—C20	1.375 (4)	C17—H17A	0.9300
N2—H2A	0.8600	C18—C19	1.393 (5)
C2—C9	1.396 (5)	C18—H18A	0.9300
C2—C3	1.436 (5)	C20—C21	1.454 (5)
O3—C21	1.327 (4)	C22—C23	1.489 (7)
O3—C22	1.456 (5)	C22—H22A	0.9700
C3—C4	1.409 (5)	C22—H22B	0.9700
C3—C8	1.415 (5)	C23—H23A	0.9600
O4—C21	1.212 (4)	C23—H23B	0.9600
C4—C5	1.372 (5)	C23—H23C	0.9600
C4—H4A	0.9300	C24—C25	1.389 (5)
C5—C6	1.390 (6)	C24—C29	1.396 (5)
C5—H5A	0.9300	C25—C26	1.388 (5)
C6—C7	1.369 (6)	C25—H25A	0.9300
C6—H6A	0.9300	C26—C27	1.358 (6)
C7—C8	1.405 (5)	C26—H26A	0.9300
C7—H7A	0.9300	C27—C28	1.380 (6)
C9—C10	1.457 (5)	C28—C29	1.380 (5)
C11—C12	1.467 (6)	C28—H28A	0.9300
C11—H11A	0.9700		
			
C8—N1—C9	109.6 (3)	C14—C13—C1	129.2 (3)
C8—N1—H1A	125.2	C15—C14—C19	117.7 (3)
C9—N1—H1A	125.2	C15—C14—C13	135.6 (3)
C2—C1—C24	111.6 (3)	C19—C14—C13	106.7 (3)
C2—C1—C13	113.6 (3)	C16—C15—C14	118.8 (4)
C24—C1—C13	113.4 (3)	C16—C15—H15A	120.6
C2—C1—H1B	105.8	C14—C15—H15A	120.6
C24—C1—H1B	105.8	C15—C16—C17	121.7 (4)
C13—C1—H1B	105.8	C15—C16—H16A	119.1
C10—O2—C11	118.2 (3)	C17—C16—H16A	119.1
C19—N2—C20	109.6 (3)	C18—C17—C16	121.4 (4)
C19—N2—H2A	125.2	C18—C17—H17A	119.3
C20—N2—H2A	125.2	C16—C17—H17A	119.3
C9—C2—C3	105.7 (3)	C17—C18—C19	117.9 (4)
C9—C2—C1	125.0 (3)	C17—C18—H18A	121.0
C3—C2—C1	129.3 (3)	C19—C18—H18A	121.0
C21—O3—C22	115.8 (3)	N2—C19—C18	129.6 (3)
C4—C3—C8	117.6 (3)	N2—C19—C14	108.0 (3)
C4—C3—C2	135.5 (3)	C18—C19—C14	122.5 (3)
C8—C3—C2	106.9 (3)	N2—C20—C13	109.0 (3)
C5—C4—C3	119.1 (4)	N2—C20—C21	117.0 (3)
C5—C4—H4A	120.5	C13—C20—C21	134.0 (3)
C3—C4—H4A	120.5	O4—C21—O3	122.9 (3)
C4—C5—C6	122.2 (4)	O4—C21—C20	123.3 (3)
C4—C5—H5A	118.9	O3—C21—C20	113.8 (3)
C6—C5—H5A	118.9	O3—C22—C23	107.4 (4)
C7—C6—C5	121.2 (4)	O3—C22—H22A	110.2
C7—C6—H6A	119.4	C23—C22—H22A	110.2
C5—C6—H6A	119.4	O3—C22—H22B	110.2
C6—C7—C8	117.2 (4)	C23—C22—H22B	110.2
C6—C7—H7A	121.4	H22A—C22—H22B	108.5
C8—C7—H7A	121.4	C22—C23—H23A	109.5
N1—C8—C7	129.0 (3)	C22—C23—H23B	109.5
N1—C8—C3	108.2 (3)	H23A—C23—H23B	109.5
C7—C8—C3	122.8 (4)	C22—C23—H23C	109.5
N1—C9—C2	109.6 (3)	H23A—C23—H23C	109.5
N1—C9—C10	118.8 (3)	H23B—C23—H23C	109.5
C2—C9—C10	131.6 (3)	C25—C24—C29	116.6 (3)
O1—C10—O2	123.8 (3)	C25—C24—C1	123.2 (3)
O1—C10—C9	122.4 (3)	C29—C24—C1	120.2 (3)
O2—C10—C9	113.7 (3)	C26—C25—C24	121.2 (4)
O2—C11—C12	107.0 (3)	C26—C25—H25A	119.4
O2—C11—H11A	110.3	C24—C25—H25A	119.4
C12—C11—H11A	110.3	C27—C26—C25	120.0 (4)
O2—C11—H11B	110.3	C27—C26—H26A	120.0
C12—C11—H11B	110.3	C25—C26—H26A	120.0
H11A—C11—H11B	108.6	C26—C27—C28	121.3 (4)
C11—C12—H12A	109.5	C26—C27—Cl1	120.4 (4)
C11—C12—H12B	109.5	C28—C27—Cl1	118.3 (3)
H12A—C12—H12B	109.5	C29—C28—C27	118.0 (4)
C11—C12—H12C	109.5	C29—C28—H28A	121.0
H12A—C12—H12C	109.5	C27—C28—H28A	121.0
H12B—C12—H12C	109.5	C28—C29—C24	122.9 (4)
C20—C13—C14	106.8 (3)	C28—C29—Cl2	118.1 (3)
C20—C13—C1	124.0 (3)	C24—C29—Cl2	119.0 (3)
			
C24—C1—C2—C9	−156.0 (3)	C13—C14—C15—C16	178.9 (4)
C13—C1—C2—C9	74.3 (4)	C14—C15—C16—C17	−0.6 (7)
C24—C1—C2—C3	23.5 (5)	C15—C16—C17—C18	1.1 (8)
C13—C1—C2—C3	−106.2 (4)	C16—C17—C18—C19	−1.1 (8)
C9—C2—C3—C4	178.7 (4)	C20—N2—C19—C18	179.4 (4)
C1—C2—C3—C4	−0.9 (7)	C20—N2—C19—C14	−0.5 (4)
C9—C2—C3—C8	0.3 (4)	C17—C18—C19—N2	−179.2 (4)
C1—C2—C3—C8	−179.3 (3)	C17—C18—C19—C14	0.7 (7)
C8—C3—C4—C5	1.4 (6)	C15—C14—C19—N2	179.6 (3)
C2—C3—C4—C5	−176.9 (4)	C13—C14—C19—N2	0.6 (4)
C3—C4—C5—C6	−0.5 (7)	C15—C14—C19—C18	−0.3 (6)
C4—C5—C6—C7	−0.6 (7)	C13—C14—C19—C18	−179.4 (4)
C5—C6—C7—C8	0.7 (6)	C19—N2—C20—C13	0.2 (4)
C9—N1—C8—C7	−176.9 (4)	C19—N2—C20—C21	−178.2 (3)
C9—N1—C8—C3	1.5 (4)	C14—C13—C20—N2	0.1 (4)
C6—C7—C8—N1	178.5 (4)	C1—C13—C20—N2	179.2 (3)
C6—C7—C8—C3	0.3 (6)	C14—C13—C20—C21	178.2 (4)
C4—C3—C8—N1	−179.8 (3)	C1—C13—C20—C21	−2.7 (6)
C2—C3—C8—N1	−1.1 (4)	C22—O3—C21—O4	0.0 (5)
C4—C3—C8—C7	−1.3 (5)	C22—O3—C21—C20	180.0 (3)
C2—C3—C8—C7	177.5 (3)	N2—C20—C21—O4	4.9 (5)
C8—N1—C9—C2	−1.4 (4)	C13—C20—C21—O4	−173.1 (4)
C8—N1—C9—C10	176.0 (3)	N2—C20—C21—O3	−175.1 (3)
C3—C2—C9—N1	0.6 (4)	C13—C20—C21—O3	7.0 (6)
C1—C2—C9—N1	−179.7 (3)	C21—O3—C22—C23	−179.8 (4)
C3—C2—C9—C10	−176.2 (3)	C2—C1—C24—C25	−110.9 (4)
C1—C2—C9—C10	3.4 (6)	C13—C1—C24—C25	18.9 (5)
C11—O2—C10—O1	−4.5 (5)	C2—C1—C24—C29	69.4 (4)
C11—O2—C10—C9	173.4 (3)	C13—C1—C24—C29	−160.8 (3)
N1—C9—C10—O1	3.8 (5)	C29—C24—C25—C26	−2.0 (5)
C2—C9—C10—O1	−179.5 (4)	C1—C24—C25—C26	178.3 (3)
N1—C9—C10—O2	−174.1 (3)	C24—C25—C26—C27	0.0 (6)
C2—C9—C10—O2	2.5 (5)	C25—C26—C27—C28	1.5 (6)
C10—O2—C11—C12	−167.2 (3)	C25—C26—C27—Cl1	−178.8 (3)
C2—C1—C13—C20	−162.4 (3)	C26—C27—C28—C29	−0.9 (7)
C24—C1—C13—C20	68.8 (4)	Cl1—C27—C28—C29	179.4 (3)
C2—C1—C13—C14	16.4 (5)	C27—C28—C29—C24	−1.2 (7)
C24—C1—C13—C14	−112.3 (4)	C27—C28—C29—Cl2	−179.8 (3)
C20—C13—C14—C15	−179.2 (4)	C25—C24—C29—C28	2.6 (6)
C1—C13—C14—C15	1.8 (7)	C1—C24—C29—C28	−177.7 (4)
C20—C13—C14—C19	−0.4 (4)	C25—C24—C29—Cl2	−178.8 (3)
C1—C13—C14—C19	−179.4 (3)	C1—C24—C29—Cl2	0.9 (5)
C19—C14—C15—C16	0.2 (6)		

### Hydrogen-bond geometry (Å, º)

Cg1 and Cg5 are the centroids of the N1,C8,C3,C2,C9 and C24–C29 rings respectively.

**Table:**

*D*—H···*A*	*D*—H	H···*A*	*D*···*A*	*D*—H···*A*
N1—H1*A*···O1^i^	0.86	2.07	2.864 (4)	152
N2—H2*A*···O4^ii^	0.86	2.04	2.871 (4)	161
C11—H11*A*···Cl1^iii^	0.97	2.81	3.731 (5)	158
C4—H4*A*···*Cg*5	0.93	2.77	3.516 (4)	137
C15—H15*A*···*Cg*1	0.93	2.72	3.476 (5)	139

Symmetry codes: (i) −*x*, −*y*+1, −*z*; (ii) −*x*+1, −*y*+2, −*z*; (iii) *x*−1, −*y*+3/2, *z*−1/2.
